# Developmental and temporal changes in petunia petal transcriptome reveal scent-repressing plant-specific RING–kinase–WD40 protein

**DOI:** 10.3389/fpls.2023.1180899

**Published:** 2023-06-08

**Authors:** Ekaterina Shor, Oded Skaliter, Elad Sharon, Yaarit Kitsberg, Dominika Bednarczyk, Shane Kerzner, Danny Vainstein, Yuval Tabach, Alexander Vainstein

**Affiliations:** ^1^ The Robert H. Smith Institute of Plant Sciences and Genetics in Agriculture, The Hebrew University of Jerusalem, Rehovot, Israel; ^2^ The Institute for Medical Research, Israel-Canada, Hadassah Medical School, The Hebrew University of Jerusalem, Jerusalem, Israel; ^3^ School of Computer Science, Tel Aviv University, Tel Aviv, Israel

**Keywords:** transcriptome, daytime, flower development, regulation, petunia scent

## Abstract

In moth-pollinated petunias, production of floral volatiles initiates when the flower opens and occurs rhythmically during the day, for optimal flower–pollinator interaction. To characterize the developmental transcriptomic response to time of day, we generated RNA-Seq databases for corollas of floral buds and mature flowers in the morning and in the evening. Around 70% of transcripts accumulating in petals demonstrated significant changes in expression levels in response to the flowers’ transition from a 4.5-cm bud to a flower 1 day postanthesis (1DPA). Overall, 44% of the petal transcripts were differentially expressed in the morning vs. evening. Morning/evening changes were affected by flower developmental stage, with a 2.5-fold larger transcriptomic response to daytime in 1DPA flowers compared to buds. Analyzed genes known to encode enzymes in volatile organic compound biosynthesis were upregulated in 1DPA flowers vs. buds—in parallel with the activation of scent production. Based on analysis of global changes in the petal transcriptome, PhWD2 was identified as a putative scent-related factor. PhWD2 is a protein that is uniquely present in plants and has a three-domain structure: RING–kinase–WD40. Suppression of *PhWD2* (termed *UPPER* - *Unique Plant PhEnylpropanoid Regulator*) resulted in a significant increase in the levels of volatiles emitted from and accumulated in internal pools, suggesting that it is a negative regulator of petunia floral scent production.

## Introduction

For angiosperms that are dependent on animal-mediated pollination, traits that make flowers conspicuous to pollinators, such as pigments and scent, are crucial for reproduction (Raguso, 2008; Sheehan et al., 2012; Frachon et al., 2021). Pigmentation of the corolla and formation of the scent-production machinery are coordinated with flower development. In petunia, anthocyanin pigment production occurs in buds, whereas volatile production initiates at flower opening (Weiss, 2000; Spitzer-Rimon et al., 2010; Cna’ani et al., 2017; Ravid et al., 2017). The phytohormone gibberellin (GA) is involved in this regulation: while biosynthesis of the former is induced by GA, production of the latter is suppressed by it. Accordingly, GA concentration is dramatically reduced at anthesis in parallel to the initiation of scent production (Ravid et al., 2017).

In addition to developmental regulation, scent is diurnally regulated, with

emission coinciding with pollinator activity (Hoballah et al., 2005; Bloch et al., 2017; Chapurlat et al., 2018; Fenske et al., 2018). *Petunia* x *hybrida* cvs. Mitchell and P720, often used in floral scent research, and their wild ancestor *Petunia axillaris*, are pollinated by a night-active moth and emit scent at night (Hoballah et al., 2005; Spitzer-Rimon et al., 2010; Fenske et al., 2015). Floral scent production has been shown to be under the control of the circadian clock, and the expression profiles of the major scent-related biosynthetic genes exhibit oscillations throughout the 24 h cycle (Fenske et al., 2015). The circadian clock genes *LATE ELONGATED HYPOCOTYL* (*LHY*) and *GIGANTEA* (*GI*) have been implicated in the regulation of petunia floral volatile production (Fenske et al., 2015; Brandoli et al., 2020). However, no study has focused on global changes in the petal transcriptome during the day.

A number of studies on the mechanisms of biosynthesis, metabolism and emission of floral scent compounds have been conducted on *Petunia* x *hybrida* (Muhlemann et al., 2014; Lynch and Dudareva, 2020; Skaliter et al., 2022). The diversity of floral volatiles found in petunia petals is represented mainly by benzenoids (benzyl alcohol, benzaldehyde, benzyl benzoate, methyl benzoate), phenylpropenes (eugenol, isoeugenol) and phenylpropanoid-related scent compounds (phenylacetaldehyde, phenylethyl alcohol, phenylethyl acetate, phenylethyl benzoate) synthesized from phenylalanine. Most enzymes and biochemical steps in this pathway leading to the production of volatile organic compounds (VOCs) have been characterized (Muhlemann et al., 2014; Huang et al., 2022; Skaliter et al., 2022).

Over the last two decades, several scent-related R2R3-MYB family transcription factors have been discovered: ODORANT1 (ODO1), EMISSION OF BENZENOIDS I (EOBI), EOBII, EOBV and PhMYB4 (Verdonk et al., 2005; Spitzer-Rimon et al., 2010; Colquhoun et al., 2011; Spitzer-Rimon et al., 2012). EOBII was shown to upregulate *ODO1* and *EOBI*, thereby activating VOC biosynthesis. Transcription of *ODO1* is also activated by EOBI and this activation is negatively affected by ETHYLENE RESPONSE FACTOR (PhERF6), the latter shown to interact with EOBI (Liu et al., 2017). In addition to the identification of these regulators, the path of volatiles from biosynthesis to their emission into the environment, and the genes controlling their transportation, are beginning to be unveiled. Suppression of *PH4* and adenosine triphosphate-binding cassette (ABC) transporter (PhABCG1) led to reduced volatile emission levels with a concomitant increase in their internal pools. Although PH4’s mode of action is unknown, PhABCG1 was found to facilitate transportation of phenylpropanoid VOCs through the plasma membrane (Cna’ani et al., 2015; Adebesin et al., 2017). VOC emission is also controlled by wax transporters PhABCG11 and PhABCG12, which affect cuticle thickness (Liao et al., 2021).

WD40-repeat proteins comprise a diversified superfamily of regulatory proteins with a common structural feature of a 40-amino acid residue unit typically ending in tryptophan (W)–aspartic acid (D) (Neer et al., 1994). They are highly abundant in eukaryotic proteomes, serving as platforms for protein–protein interactions to regulate numerous cell processes, including specialized metabolism such as carotenoid and flavonoid biosynthesis (Zimmermann et al., 2004; Ramsay and Glover, 2005; Baudry et al., 2006). Morer recently, the involvement of WD protein Tannin 1 in regulating the production of fatty acid-derived volatiles in sorghum was demonstrated (Xie et al., 2019). Furthermore, in petunia flowers, the WD40 protein ANTHOCYANIN 11 (AN11) acts as a major hub that controls corolla pigmentation *via* a complex network of interchanging MYB, bHLH and WRKY partners that transcriptionally regulates genes encoding enzymes of the vacuolar acidification and anthocyanin-biosynthesis pathways (de Vetten et al., 1997; Quattrocchio et al., 2006; Albert et al., 2011; Albert et al., 2014; Verweij et al., 2016; Zhang et al., 2019). Anthocyanin production was shown to be interlinked with the biosynthesis of phenylalanine-derived volatiles (Zuker et al., 2002; Zvi et al., 2008; Cna’ani et al., 2015), yet involvement of WD40 proteins in benzenoid/phenylpropanoid floral VOC production has not been documented.

Here we detailed transcriptomes of petunia petals in buds and mature flowers at the beginning and end of the day. We revealed that compared to buds, mature flowers show a higher transcriptomic response to the time of day, with enrichment in pathways involved in central biological processes and secondary metabolism. Detailed transcriptome analyses led to the identification of a WD40 domain protein, termed here PhWD2, that acts as a negative regulator of VOC production in petunia.

## Materials and methods

### Plant material

*Petunia* x *hybrida* line Mitchell diploid (W115), used to generate transcriptome databases, and *P. hybrida* line P720 plants were grown in a greenhouse under 25°C day/20°C night temperatures with a 16-h light/8-h dark photoperiod (lights on at 0600 h/lights off at 2200 h).

### Generation of the transcriptome databases

For RNA-Seq analysis, samples were collected from petals of petunia ‘W115’ plants in the morning at 1000 h and in the evening at 1900 h from buds (4.5 cm in height) and opened flowers at 1 day postanthesis (1DPA). RNA extraction was performed using the RNeasy Plant Mini Kit (Qiagen) according to the manufacturer’s instructions. For DNase treatment, Invitrogen DNA-free kit DNase Treatment and Removal (Thermo Fisher Scientific) were used. The cDNA was synthesized using RNA, oligo(dT) primers and ImProm‐II (Promega) reverse transcriptase according to the manufacturer’s instructions. Three biological replicates for each type of sample were used for sequencing. 100 bp single-end reads were sequenced on Novaseq SP of an Illumina NovaSeq. The output was ~21 million reads per sample. Briefly, the polyA fraction (mRNA) was purified from 500 ng of total input RNA, followed by fragmentation and the generation of double-stranded cDNA. After Agencourt Ampure XP bead cleanup (Beckman Coulter), end repair, A base addition, adapter ligation and PCR amplification steps were performed. Libraries were quantified by Qubit (Thermo Fisher Scientific) and TapeStation (Agilent). Sequencing was done on a NextSeq High Output Kit (75 cycles) (Illumina; single-read sequencing). Poly-A/T stretches and Illumina adapters were trimmed from the reads using cutadapt (Martin, 2011); resulting reads shorter than 30 bp were discarded. Reads were mapped to the *Petunia axillaris* reference genome (https://solgenomics.net/ftp/genomes/Petunia_axillaris/Peaxi162annotation_v4.gff) using STAR (Dobin et al., 2013), supplied with gene annotations downloaded from Sol Genomics Network (https://solgenomics.net/) (and with EndToEnd option and outFilterMismatchNoverLmax set to 0.04). Reads with the same UMI were removed using the PICARD MarkDuplicate tool with the BARCODE_TAG parameter. Expression levels for each gene were quantified with htseq-count (Anders et al., 2015), using the above gff file. Normalization of raw counts and identification of differentially expressed genes (DEGs) were performed using DESeq2 (Love et al., 2014) with the betaPrior, cooksCutoff and independent Filtering parameters set to False. Raw *P* values were adjusted for multiple testing using the Benjamini–Hochberg Procedure. The pipeline was run using snakemake (Köster and Rahmann, 2012). Transcripts with *P* ≤ 0.05, absolute value of log_2_ fold change (FC) ≥ 0.585 and normalized count ≥ 30 were considered to be DEGs. Genes with log_2_ FC ≥ 0.585 were considered to be upregulated, and those with log_2_ FC ¾ -0.585 to be downregulated.

### Virus-induced gene silencing

Suppression of *PhWD*s in petunia ‘P720’ flowers was performed using VIGS as described previously (Spitzer et al., 2007). Briefly, to generate Tobacco rattle virus TRV2 with a fragment of *CHALCONE SYNTHASE* (pTRV2-CHS) as a marker for viral spread (Spitzer et al., 2007) and fragments of the target WD40s *PhWD1*, *PhWD2* or *PhWD3*, corresponding fragments were amplified from the 3’ untranslated region of cDNA by PCR using the primers shown in **Supplementary Table S1**. Sequences of the amplified fragments were analyzed by VIGS Tool on the Sol Genomics Network (https://vigs.solgenomics.net/) to verify the absence of putative off-targets. These plasmids were introduced into *Agrobacterium tumefaciens* (AGLO) and mixed with agrobacterium carrying pTRV1 in a 1:1 ratio in inoculation solution containing 200 µM acetosyringone and 10 mM MgCl_2_. The mixture was applied to the cut surface of the petunia ‘P720’ stem after removing the apical meristem. Plants were grown in the greenhouse for ca. 1 month and flowers with white areas (indicating viral infection) were used for analyses. Localized transient suppression of *PhWD2* was performed as described previously (Shor et al., 2023). Petals of flowers at anthesis were injected with the inoculation solution containing agrobacteria carrying pTRV1 and TRV2-CHS-PhWD2 or TRV2-CHS (control). After 48 h, inoculated petal regions were used for localized headspace extraction of volatiles from internal pools and for RNA extraction. The experiments were performed in two to three biological repeats with similar results.

### Collection of emitted VOCs and GC-MS analysis

For analysis of emitted floral scent compounds, VOCs were collected by dynamic headspace from 1DPA flowers placed in beakers filled with tap water and into jars (3 flowers/jar) or by localized headspace (Skaliter et al., 2021) from the agroinfiltrated petal regions of 2DPA flowers. Volatile collection was performed for 24 h by applying vacuum and using columns made of glass tubes containing 100 mg Porapak Type Q polymer and 100 mg 20/40-mesh activated charcoal, held in place with plugs of silanized glass wool. Trapped volatiles were eluted with 1.5 mL hexane and 0.5 mL acetone; 0.24 µg isobutyl benzene was added to each sample as an internal standard prior to GC-MS analysis. GC-MS analysis of VOCs was performed as described previously (Skaliter et al., 2021). The experiments were performed in three to four biological repeats with similar results.

### Extraction of floral scent compounds from the internal pools

To determine the levels of VOCs in the internal pool of petal tissues, TRV-infected petal regions were collected at 2000 h when VOCs in the pools are at the midpoint between their lowest and highest levels (Cna’ani et al., 2017). Tissues were ground in liquid nitrogen and VOCs were extracted for 2 h in hexane (4 mL/sample), containing 2 μg isobutylbenzene as the internal standard. After the spin, supernatant was subjected to GC-MS analysis. The experiments were performed in three biological repeats with similar results.

### Real-time PCR analysis

For epidermis samples, adaxial epidermal tissues were peeled under a microscope (Olympus SZ-40) as described previously (Skaliter et al., 2021). For RNA extraction from buds, petals and epidermal tissues, the Tri‐Reagent Kit (Sigma‐Aldrich) was used. The obtained RNA was treated with RNase‐free DNase I (Thermo Fisher Scientific) prior to cDNA synthesis using ImProm‐II (Promega) reverse transcriptase and oligo(dT) primers. Two-step real‐time quantitative PCR (qRT-PCR) was performed on a Rotor‐Gene Q cycler (Qiagen) with 2X qPCRBIO SyGreen Blue Mix Hi-ROX (PCR Biosystems) according to the manufacturer’s protocol. Raw transcript level data were normalized to *EF1α* or *actin*. Quantification calculations were carried out using the 2^−ΔΔCT^ formula as described previously (Nozue et al., 2007), unless otherwise stated. The primers are shown in **Supplementary Table S2**. The experiments were performed in two to three biological repeats with similar results.

### Bioinformatics analysis and tools

Heatmaps were generated using seaborn library and the complex heatmap package in R (Gu et al., 2016). Kyoto Encyclopedia of Genes and Genomes (KEGG) and gene ontology (GO) enrichment analyses were performed using ShinyGO (http://bioinformatics.sdstate.edu/go/). Putative Arabidopsis homologs of petunia genes, predicted by reciprocal blast, were submitted as input and a false discovery rate (FDR) cutoff of 0.05 was applied.

Conservation analysis was performed as in Braun et al. (2020). Data for proteomes of selected organisms were generated by integrating three databases: Ensembl (Yates et al., 2020), NCBI Genomes Refseq (Sayers et al., 2020) and Uniprot “reference proteomes” (Chen et al., 2011). The inferred proteomes were merged at the species level and each organism was filtered from duplicated sequences, resulting in 1952 eukaryotic organisms. Conservation score was calculated based on the bit-score received from BLASTP (Altschul et al., 1990) between the protein sequence of PhWD2 and the top result for each organism. To reduce the influence of random matches, low BLASTP bit-scores (e-value > 10^-5^) were assigned a value of 0. The conservation score represents the ratio of the observed BLASTP score and the best possible BLASTP score of the same length (the self-similarity score) and is defined as Pab/Paa, where Pab is the best BLASTP bit-score between PhWD2 (“a”) and a protein from the collected proteome (“b”). Paa is the self-similarity score of the petunia protein “a” when blasted against itself. Conservation scores below 10% were assigned a value of 0.

For domain analysis, protein sequences of the Viridiplantae clade were analyzed using the NCBI Batch Conserved Domains-Search Tool (Marchler-Bauer and Bryant, 2004) with an e-value cutoff of 0.01.

## Results

### Significant perturbations in the petunia corolla transcriptome depend on flower developmental stage

**Figure 1:**
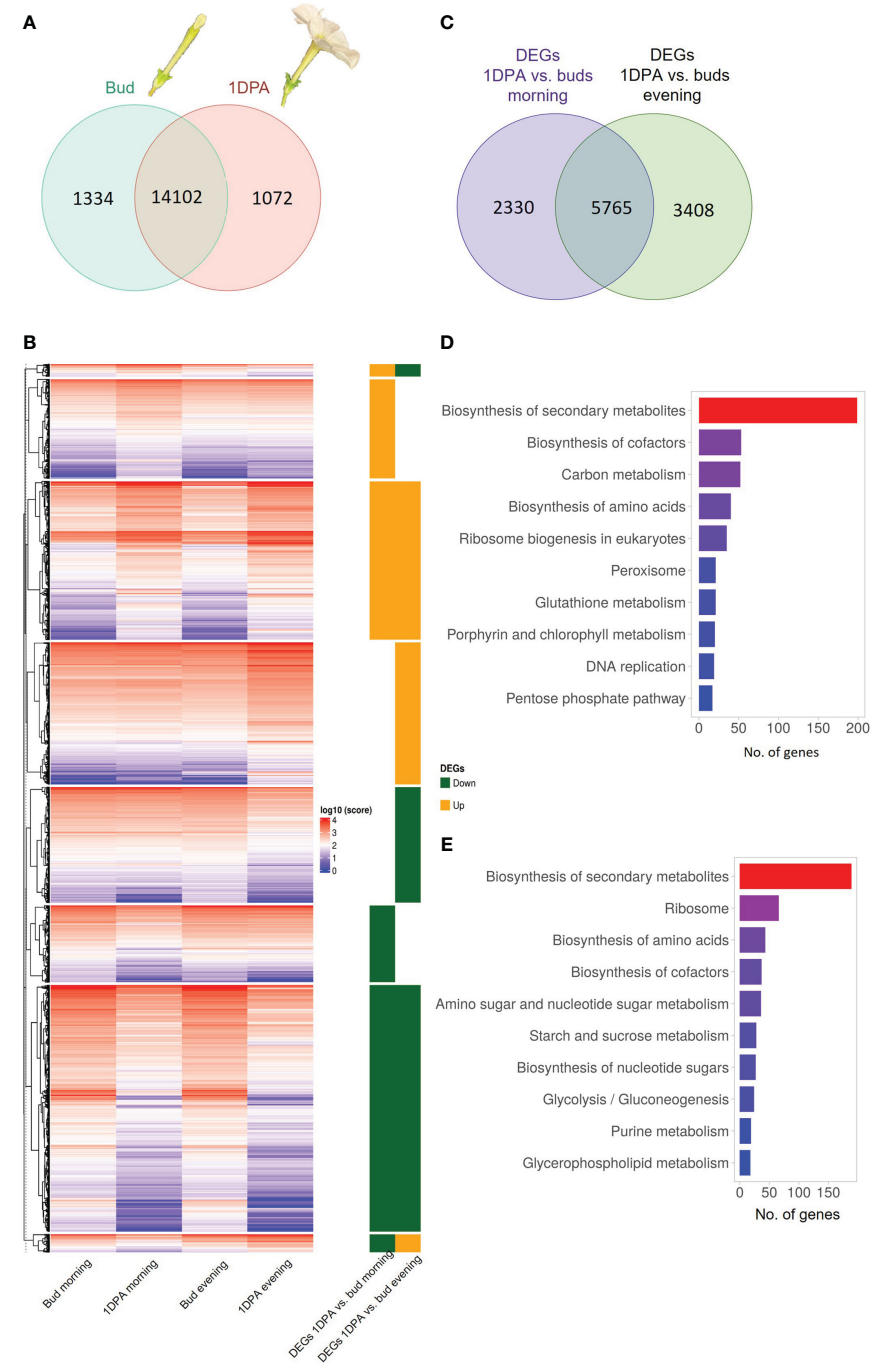
Transcriptomic profile of petals of petunia buds and 1 day postanthesis (1DPA) flowers. **(A)** Venn diagram displaying number of genes expressed in petals of buds and/or 1DPA flowers. **(B)** Expression levels of differentially expressed genes (DEGs) in the corolla of 1DPA flowers vs. buds. Average normalized counts of three biological repeats for each gene are plotted. Right panel: 1DPA vs. bud DEGs that are upregulated (“Up”) and downregulated (“Down”) in the morning and in the evening. Data are shown in log_10_ scale. **(C)** Venn diagram displaying the number DEGs for 1DPA vs. bud comparison in the morning and evening. **(D, E)** KEGG enrichment for **(D)** upregulated and **(E)** downregulated 1DPA vs. bud DEGs.

Flowers of *Petunia* x *hybrida* begin to produce scent when the flower unfurls. This scent production is rhythmic—increasing toward nighttime Indeed, expression of transcripts encoding scent-related genes often coincides with these developmental and diurnal patterns (Fenske et al., 2015). Therefore, we generated a transcriptomic database to characterize and integrate these two patterns. This database can further serve as a platform for the identification of novel scent-related genes. We performed RNA-Seq analysis on corollas of 4.5-cm buds and 1DPA flowers of cv. Mitchell at two time points: morning (1000 h) and evening (1900 h). The analysis identified 21,201 transcripts representing ca. 64% of all *P. axillaris* protein-coding genes (Bombarely et al., 2016). Of the 15,174 genes expressed in 1DPA petals (normalized raw count ≥ 30), 1072 genes were not expressed in buds (“1DPA-specific”), whereas of the 15,436 transcripts expressed in buds, 1334 were bud-specific (**Figure 1A**). GO analysis of 1DPA-specific petal genes revealed enrichment in reproduction-related biological processes. The group of bud-specific genes was enriched in protein phosphorylation, cell wall organization and tissue development biological processes (**Supplementary Figures S1A** and **B**, **Supplementary Dataset S1**).

The obtained transcriptomes were subjected to analysis for differential gene expression. Genes with an absolute FC value _ 1.5 and *p*-value ≤ 0.05 were designated as DEGs. The expression levels of all 1DPA vs. bud DEGs are shown as normalized counts on a heatmap (**Figure 1B**, **Supplementary Dataset S2**). Of the 16,508 transcripts expressed in petals, 11,503 displayed significant changes in expression level between buds and 1DPA open flowers; 20.3% of them were affected by flower developmental stage only in the morning, 29.6% only in the evening, and 50.1% at both time points (**Figure 1C**, **Supplementary Dataset S3**). Of the 5693 DEGs that were upregulated in 1DPA flowers compared to buds (in the morning and/or in the evening), 1479 were upregulated only in the morning, and 2115 only in the evening. There were 6212 DEGs that were downregulated in 1DPA flowers compared to buds in the morning and/or in the evening, with 1253 downregulated only in the morning, and 1695 only in the evening (**Figure 1B**, **Supplementary Figure S1C**, **Supplementary Dataset S3**).

**Figure 2:**
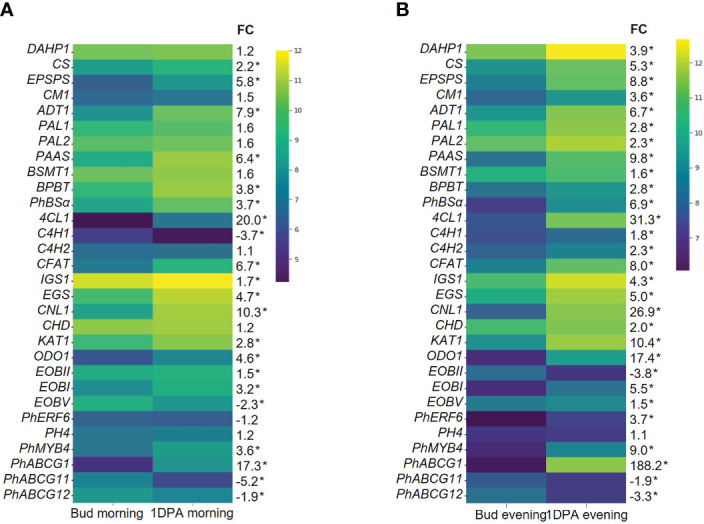
Expression levels of scent-related genes in corollas of petunia floral buds and 1 day postanthesis (1DPA) flowers. Transcript levels detected by RNA-Seq **(A)** in the morning (1000 h) and **(B)** in the evening (1900 h). Natural logarithms of average normalized counts for three biological replicates are plotted (n = 3). FC, fold change in average normalized counts for 1DPA vs. bud. *Transcripts detected as differentially expressed genes (DEGs) in 1DPA vs. bud comparison (*P* ≤ 0.05, |log_2_FC| ≥ 0.585, normalized count ≥ 30). Abbreviations: DAHP, deoxy-D-arabino-heptulosonate-7-phosphate; EPSPS, 5-enol-pyruvylshikimate-3-phosphate synthase; CS, chorismate synthase; CM, chorismate mutase; ADT, arogenate dehydratase; PAL, L-phenylalanine ammonia lyase; PAAS, phenylacetaldehyde synthase; BSMT, S-adenosyl-L-methionine:benzoic acid/salicylic acid carboxyl methyltransferase; BPBT, benzoyl-CoA:benzyl alcohol/2-phenylethanol benzoyltransferase; 4CL, 4-coumarate:CoA ligase; PhBSα, benzaldehyde synthase; C4H, cinnamate-4-hydroxylase; CFAT, coniferyl alcohol acetyltransferase; IGS, isoeugenol synthase; EGS, eugenol synthase; KAT, 3-ketoacyl-CoA thiolase; CNL, cinnamoyl-CoA ligase; CHD, cinnamoyl-CoA hydratase-dehydrogenase; EOBI/II/V, emission of benzenoids I/II/V; ODO1, odorant 1; PhERF6, ethylene response factor 6.

KEGG analysis of all DEGs that were upregulated in 1DPA flowers vs. buds revealed highest enrichment in secondary metabolite-biosynthesis pathways (**Figure 1D**, **Supplementary Dataset S3**). Enzymes encoded by these genes included those leading to the production of benzenoid/phenylpropanoid volatiles: enolpyruvate-shikimate-3-phosphate synthase (EPSPS), chorismate synthase (CS), chorismate mutase (CM1), L-phenylalanine ammonia lyase (PAL1), 4-coumarate:CoA ligase (4CL), benzaldehyde dehydrogenase (BALDH1) were upregulated in both the morning and evening; deoxy-D-arabino-heptulosonate-7-phosphate (DAHP1), arogenate dehydratase (ADT3), PAL2 and 3-ketoacyl-CoA thiolase (KAT2) were upregulated only in the evening, and CM2 only in the morning. These results are in line with the expected increase in expression of VOC-biosynthesis genes in parallel to the transition from bud to mature flower (Cna’ani et al., 2017). The expression levels of the characterized scent-related genes (isoform with highest expression) in buds and 1DPA flowers are shown in **Figure 2**. All of these genes, except for transcription factor *PH4*, were affected by the flower’s developmental stage. The genes encoding biosynthesis enzymes of the phenylpropanoid pathway branch, leading to the production of VOCs, were more highly expressed in 1DPA petals than in buds. This gene-expression pattern is supported by previous studies (Colquhoun et al., 2010; Maeda et al., 2010; Klempien et al., 2012; Huang et al., 2022) and is in accordance with the initiation of VOC biosynthesis when the flower opens. Upregulation of biosynthesis genes *DAHP1*, *BENZOIC ACID/SALICYLIC ACID CARBOXYL METHYLTRANSFERASE* (*BSMT1*), *PAL1*, *PAL2*, *CINNAMOYL-COA HYDRATASE-DEHYDROGENASE* (*CHD*), *CINNAMATE-4-HYDROXYLASE* (*C4H2*) and *CM1* in 1DPA petals vs. buds was significant in the evening but not in the morning. Transcriptional regulators of VOC biosynthesis *ODO1*, *PhMYB4* and *EOBI* were more highly expressed in 1DPA petals than in buds in both morning and evening; *EOBII* was more highly expressed only in the morning. Wax transporters *PhABCG11* and *PhABCG12* were more highly expressed in buds than in mature flowers, coinciding with cuticle formation (Liao et al., 2021).

Scent-related genes have been shown to be regulated by GA. Patrick et al. (2021) suggested that in open flowers, as compared to buds, GA levels are low, while sensitivity to GA increases. Accordingly, in our RNA-Seq data, genes involved in GA signaling were differentially expressed in 1DPA flowers vs. buds (**Supplementary Figure S2A**). For example, gibberellin receptors *GID1B1*, *GID1B2*, *GID1B3GA* and *GID1C* were more highly expressed in 1DPA petals than in buds. The GA catabolic gene *GA2ox2a* also showed higher expression in 1DPA petals vs. buds.

KEGG analyses of DEGs with lower expression in 1DPA flowers than in buds revealed enrichment in secondary metabolite-biosynthesis pathways (**Figure 1E**, **Supplementary Dataset S2**). These included genes involved in phenylalanine biosynthesis, e.g., *DAHP3* which was downregulated in the morning and *ADT4* which was downregulated in both morning and evening; and in anthocyanin biosynthesis, e.g., *DIHYDROFLAVONOL 4-REDUCTASE* (*DFR*) and *FLAVANONE 3-HYDROXYLASE* (*F3H*) which were downregulated in the evening, and *CHALCONE ISOMERASE* (*CHI-A*) which was downregulated in the morning. Expression of the major anthocyanin biosynthesis genes is shown in **Supplementary Figure S2B**. In accordance with previous research (Patrick et al., 2021), and as also occurs in the pigmented flowers of another *P. hybrida* variety (Brugliera et al., 1999), expression of these genes was higher in buds than in 1DPA flowers. It should be noted that although the mature flowers of petunia cv. Mitchell analyzed here do not develop a colored corolla, young flower buds accumulate low but noticeable levels of pigmentation (Albert et al., 2011). Genes encoding ribosomal proteins were also KEGG-enriched (among the DEGs that were more highly expressed in buds vs. 1DPA flowers), probably reflecting a decrease in protein biosynthesis and cell division (Fujikura et al., 2009) in the petals of mature flowers. This is in agreement with the gradual switch from cell division to expansion in the corolla toward anthesis (Sood et al., 2022).

Among the DEGs in 1DPA flowers vs. buds, there were also two small groups of transcripts whose day/night expression pattern changes with flower developmental stage. These consisted of 165 and 237 genes that were, respectively, upregulated in the morning and downregulated in the evening, or vice versa (**Figure 1B**, **Supplementary Dataset S2**). For these genes, the amplitude of the changes in morning/evening mRNA levels was higher at one developmental stage than at the other, as observed for example for *HYDROXYCINNAMOYL TRANSFERASE* (*HCT*) and *C4H*; or the time of high and low expression reversed, as in case of *CINNAMOYL COA REDUCTASE* (*CCR1*).

### Temporal expression pattern of genes is affected by flower developmental stage

**Figure 3:**
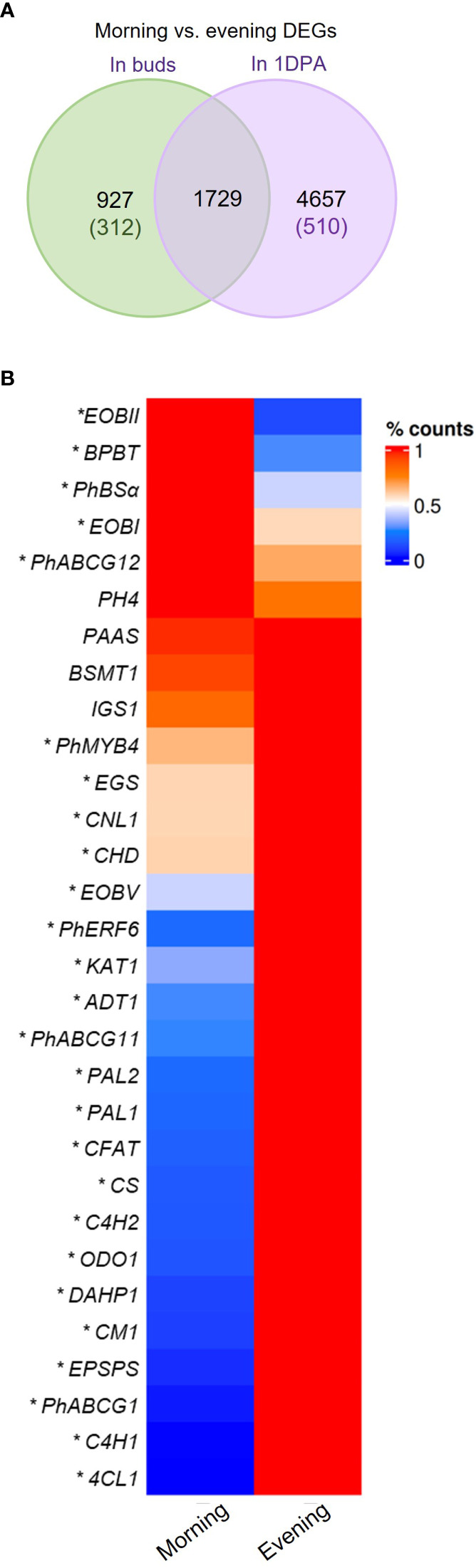
Morning vs. evening changes in expression of petunia genes. **(A)** Morning vs. evening differentially expressed genes (DEGs) in petals of buds and 1 day postanthesis (1DPA) flowers. Green and purple font indicate number of DEGs expressed only in buds or only in 1DPA flowers, respectively. **(B)** mRNA levels of petunia scent-related genes in petals of 1DPA flowers in the morning (1000 h) and in the evening (1900 h) by RNA-Seq analysis. Average counts normalized to maximum for each gene are plotted. *Transcripts detected as DEGs in morning vs. evening comparison (*P* ≤ 0.05, |log_2_FC| ≥ 0.585, normalized count ≥ 30). Abbreviations: see **Figure 2**.

Analysis of morning vs. evening (m/e) changes in mRNA levels revealed that nearly half of the genes expressed in 1DPA flowers (42%, 6386 out of 15,174 transcripts) were detected as DEGs in the m/e comparison, whereas only 17.2% (2656 out of 15,436 transcripts) of the genes expressed in buds were m/e DEGs (**Figure 3A**). This suggests that petals of 1DPA flowers display a larger transcriptomic response to diurnal changes than buds. About half of the 1DPA-specific transcripts (510 out of 1072) and only about one-quarter of the bud-specific transcripts (312 out of 1334) were m/e DEGs. Furthermore, among all of the 1DPA m/e DEGs (6386 transcripts), only 27% (1729 transcripts) were also m/e DEGs in buds, whereas almost 65% of all bud m/e DEGs (1729 out of 2656 transcripts) were also m/e DEGs in 1DPA petals, suggesting that the diurnal pattern of gene expression in petals depends on flower developmental stage (**Figure 3A**, **Supplementary Dataset S4**). About 92% (5875 transcripts) of all 1DPA m/e DEGs were expressed in both 1DPA flowers and buds. Nevertheless, around 80% of these genes were m/e DEGs only in 1DPA flowers, but not in buds (**Supplementary Figure S3A**). Interestingly, more than half of these transcripts (2478) were expressed at higher levels in buds. KEGG analysis of this group revealed enrichment in biosynthesis of secondary metabolites, ribosomal proteins, purine metabolism and biosynthesis of nucleotide sugar pathways (**Supplementary Figure S3B**, **Supplementary Dataset S4**). This may further suggest that mature flowers acquire a diurnal pattern of central biological processes as well as secondary metabolism, in line with the establishment of time-dependent flower-specific processes.

Mature flowers of *Petunia* x *hybrida* produce floral volatiles with diurnal rhythmicity. Expression levels of the major scent-related genes demonstrated m/e changes (**Figure 3B**, **Supplementary Figure S4**). Our analysis revealed patterns that were similar to those previously reported: *EPSPS*, *CM1*, *ADT1*, *PAL1,2, 4-COUMARATE : CoA LIGASE* (*4CL*), *CONIFERYL ALCOHOL ACETYLTRANSFERASE* (*CFAT*), *ODO1, PhABCG1, PhERF6* and *PhMYB4* were more highly expressed in the evening; and *EOBI*, *EOBII*, *BENZOYL-COA : BENZYL ALCOHOL/2-PHENYLETHANOL BENZOYLTRANSFERASE* (*BPBT*)*, BENZALDEHYDE SYNTHASE* (*BSα*) were more highly expressed in the morning Morning and evening levels of *PHENYLACETALDEHYDE SYNTHASE* (*PAAS*), *BSMT1, ISOEUGENOL SYNTHASE* (*IGS*) and *PH4* were not significantly different, possibly because their peak expression is at midday (Boatright et al., 2004; Colquhoun et al., 2010; Cna’ani et al., 2015; Fenske et al., 2015). The mRNA levels of the scent regulator *EOBV* and of the genes *DAHP1*, *CS*, *C4H*s, *CINNAMOYL-COA LIGASE* (*CNL1*), *CHD*, *KAT1* encoding biosynthesis enzymes, were higher in the evening than in the morning. Interestingly, the expression of wax transporters *PhABCG11* and *PhABCG12* was also affected by the time of day, but they demonstrated opposite patterns—*PhABCG11* expression was higher in the evening whereas that of *PhABCG12* was higher in the morning (**Figure 3B**). The evening expression of the genes encoding enzymes involved in the biosynthesis of the VOC precursor phenylalanine (*DAHP1*, *EPSPS*, *CS, CM1*, *ADT1*) was in agreement with high accumulation of this amino acid toward evening in petunia petals (Maeda et al., 2010).

### Screening for WD40 proteins reveals PhWD2 as a scent regulator

WD40 domain proteins are involved in numerous cellular processes, including the biosynthesis of specialized metabolites such as flavonoids—a process that is linked to scent production in petunia flowers (Zvi et al., 2008; Cna’ani et al., 2015). Furthermore, the WD40s’ involvement in the biosynthesis of fatty acid-derived VOCs has been recently reported (Xie et al., 2019). To assay the involvement of WD40 proteins in phenylpropanoid volatile production in flowers, we screened our transcriptomic databases for DEGs that are upregulated in 1DPA flowers vs. buds and that are annotated as encoding WD40 proteins; 43 genes fulfilled these criteria (**Supplementary Dataset S5**). Genes with less than 100 normalized counts in 1DPA flowers were not considered for further analyses. As scent-related genes can peak in the morning or in the evening, these WD40-encoding genes were divided into three groups according to the timing of their highest expression in mature petals: morning (4 genes), evening (6 genes), and with no change in m/e expression (21 gene). One candidate was selected from each group: *Peaxi162Scf00006g00099*, termed *PhWD1*, which has 1.5-fold higher expression in the evening compared to the morning, *Peaxi162Scf01003g00015*, termed *PhWD2*, which has 1.5-fold higher expression in the morning compared to the evening, and *Peaxi162Scf00378g00630*, termed *PhWD3*, with no change in m/e expression. The presence of WD40 repeats in the three genes was verified using the PROSITE (https://prosite.expasy.org/) and WDSPdb (Ma et al., 2019) tools; interestingly it also revealed an unusual protein structure of PhWD2.

**Figure 4:**
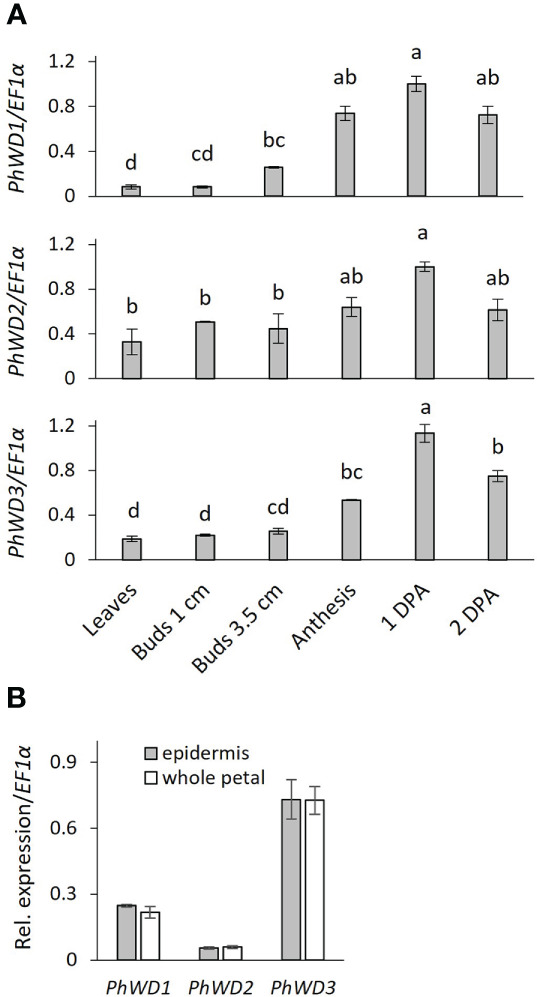
Developmental and spatial expression profiles of *PhWD1, 2* and *3*. Quantitative RT-PCR was performed on RNA extracted from: **(A)** young leaves, petals of floral buds and open flowers (anthesis, 1DPA, 2DPA). Samples were collected at 1700 h. Transcript levels of the targets were normalized to *EF1α* and then to the maximum level for all of the samples in the experiment. Significance of the differences was calculated by one-way ANOVA with post-hoc Tukey HSD test. Values with different letters are significantly different at *P* ≤ 0.05, and **(B)** petal adaxial epidermis and whole-petal tissue of 1 day postanthesis (1DPA) flowers. Samples collected at 1000 h. Relative expression was calculated by ΔCT method; *EF1α* was used as reference gene. Significance of differences was calculated using Student’s t-test. Data are means ± SEM (n = 3).

**Figure 5:**
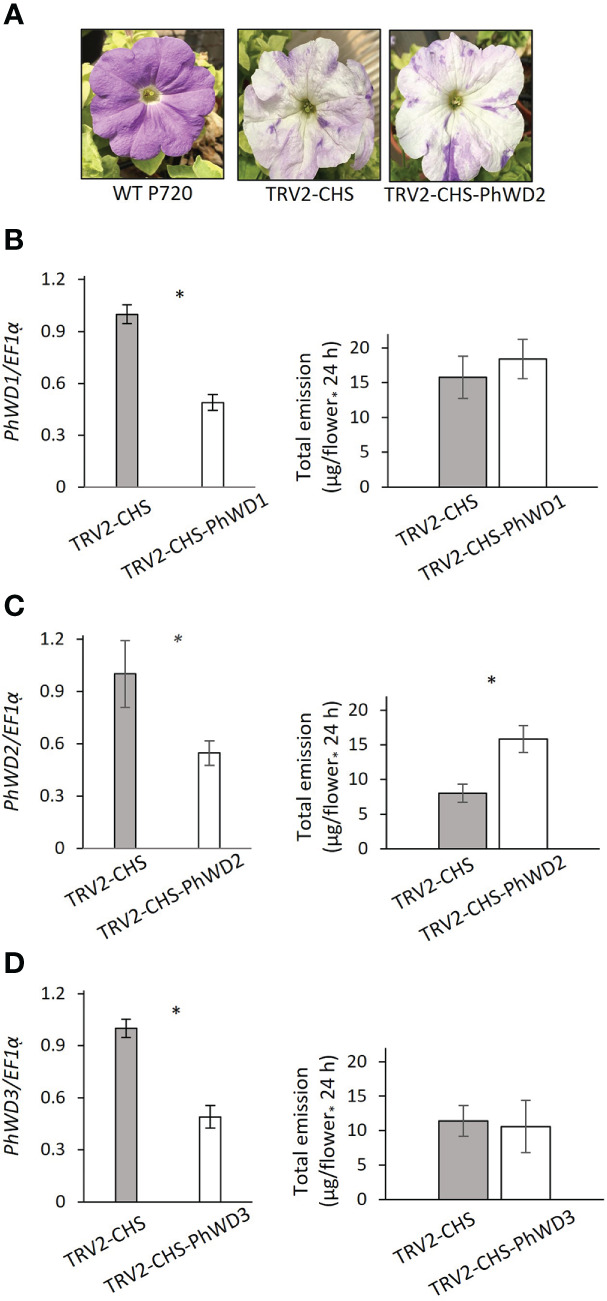
Suppression of *PhWD2* leads to increased emission levels of volatiles. **(A)** Representative flowers of *P. hybrida* line P720 wild type (WT) and after infection with TRV2-CHS and TRV2-CHS-PhWD2. Flowers were infected with **(B)** TRV2-CHS-PhWD1, **(C)** TRV2-CHS-PhWD2, **(D)** TRV2-CHS-PhWD3 or the corresponding control (TRV2-CHS). Normalized relative expression of *PhDW1*, *PhDW2* and *PhDW3*, measured by qRT-PCR, and total emitted volatile organic compounds collected by dynamic headspace following VIGS-mediated suppression are presented. *EF1α* was used as an internal reference gene. Expression levels of the targets were normalized to those in the control samples. Data are means ± SEM (n = 4–7). Significance of differences between treatments was calculated using Student’s t-test: *P ≤ 0.05.

**Figure 6:**
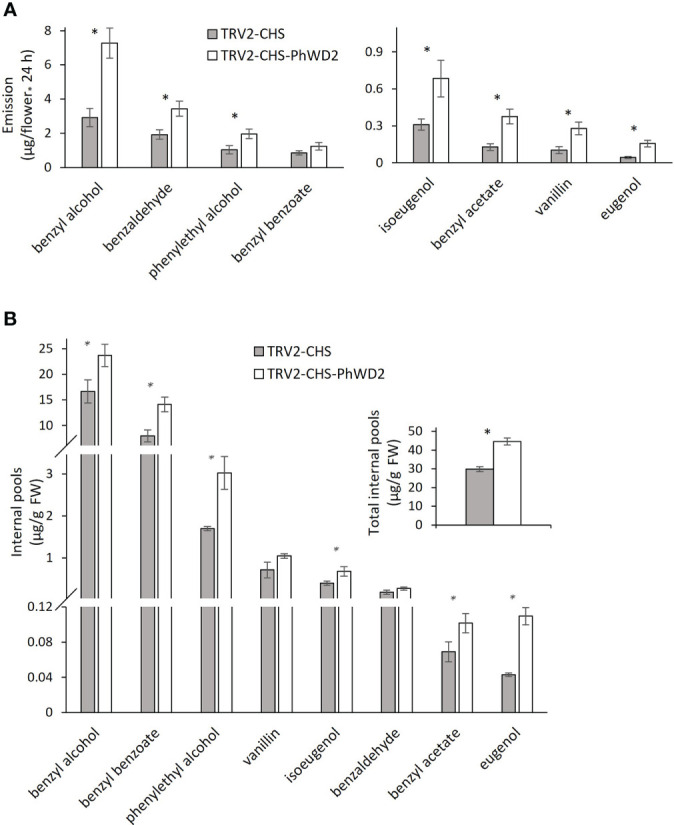
Suppression of *PhWD2* results in a marked increase in the production of individual volatile organic compounds (VOCs). *P. hybrida* line P720 plants were infected with TRV2-CHS-PhWD2 or TRV2-CHS (control). Flowers 1 day postanthesis (1DPA) were used for dynamic headspace or extraction of VOCs from internal pools followed by GC-MS analysis. Levels of individual VOCs **(A)** emitted (n = 7–8) and **(B)** accumulated in internal pools (n = 4). Inset: total VOCs accumulated in internal pools. Data are means ± SEM. Significance of differences between treatments was calculated using Student’s t-test: *P ≤ 0.05.

More detailed analysis of *PhWD1*, *PhWD2* and *PhWD3* mRNA levels throughout flower development revealed that their expression gradually increases with bud growth and flower opening, and is highest in mature flowers (**Figure 4A**). Since the adaxial petal epidermis is the main site of volatile biosynthesis (Skaliter et al., 2021), we analyzed the *PhWD*s’ expression levels in the epidermis as compared to that in the whole petals. **Figure 4B** shows that *PhWD1*, *PhWD2* and *PhWD3* are expressed in epidermis at the levels similar to those in whole-petal tissues. To test the relevance of these PhWDs to scent regulation, we suppressed the expression of each of their genes in petals of *Petunia* x *hybrida* line P720 using VIGS, as described previously (Spitzer-Rimon et al., 2013). Young petunia plants were infected with agrobacteria carrying TRV-derived plasmids TRV2-CHS-PhWD1, TRV2-CHS-PhWD2 or TRV2-CHS-PhWD3, or TRV2-CHS as a control. The *CHS* fragment was used in all of these plasmids to suppress this gene, which encodes the key enzyme of the anthocyanin pathway; this enabled visualizing the VIGS-affected petal areas (**Figure 5A**) without affecting floral VOCs production (Spitzer et al., 2007) Infected plants developed and flowered normally with no observable differences compared to control TRV2-CHS-inoculated plants. TRV-induced suppression resulted in a ca. 50% decrease in expression levels of all target *PhWD*s (**Figure 5B**). TRV2-CHS-PhWD1, TRV2-CHS-PhWD2, TRV2-CHS-PhWD3 and TRV2-CHS flowers were subjected to dynamic headspace analysis to evaluate scent phenotype. Suppression of *PhWD1* and *PhWD3* did not cause significant changes in the levels of total emitted volatiles compared to the TRV2-CHS control, whereas suppression of *PhWD2* resulted in an increase in total VOC emission (**Figure 5C**), indicating its involvement in the regulation of floral scent production. Next, we detailed the effect of *PhWD2* suppression on the emission of individual volatile compounds. The emission levels of the major petunia floral scent compounds were significantly increased in flowers with TRV-suppressed *PhWD2* (TRV2-CHS-PhWD2) compared to control TRV2-CHS flowers (**Figure 6A**), except for benzyl benzoate which was not significantly elevated. Analyses of volatiles in internal pools showed that the total content of VOCs, as well as the levels of most of the scent compounds, accumulated in the pools were higher in TRV2-CHS-PhWD2 than in the control. Vanillin and benzaldehyde were not significantly affected by *PhWD2* suppression (**Figure 6B**). Taken together, suppression of *PhWD2* led to a significant increase in the emission and accumulation of phenylpropanoid volatiles, positing PhWD2 as a negative regulator of scent production.

### PhWD2, a repressor of floral VOC production, has a unique three-domain structure

**Figure 7:**
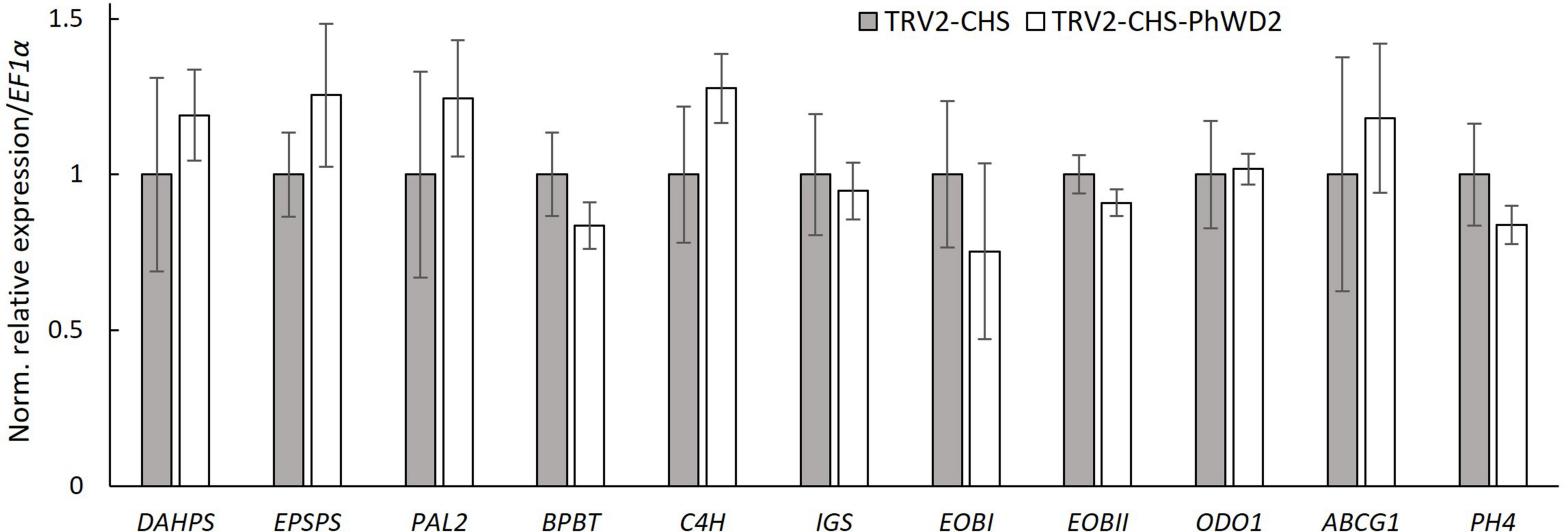
Expression of scent-related genes in petals following localized suppression of PhWD2. Petals of petunia line P720 flowers at anthesis were inoculated with *Agrobacterium* suspension carrying TRV2-CHS-PhWD2 or TRV2-CHS (control). Inoculated petal tissues were harvested from 2 days postanthesis (2DPA) flowers at 2000 h for RNA extraction followed by qRT-PCR analyses. *EF1α* was used as an internal reference gene. Expression levels of the targets were normalized to those in the control samples. Data are means ± SEM (n = 4). For statistical analysis, Student’s t-test was applied.

To further detail the VIGS-based effect of *PhWD2* on floral scent, we transiently suppressed *PhWD2* locally, in the petals of line P720 flowers. Petals were inoculated with suspensions of *Agrobacterium* carrying TRV2-CHS-PhWD2 or TRV2-CHS (control), and agroinfiltrated tissues were used for further analysis. The level of *PhWD2* in the inoculated area was ca. 2-fold lower in TRV2-CHS-PhWD2-inoculated petals than in controls (**Supplementary Figure S6A**). The total emission of volatiles measured by localized headspace analysis and the total level of VOCs accumulated in internal pools, were significantly enhanced in TRV2-CHS-PhWD2-inoculated areas as compared to the control (**Supplementary Figure S6B, C**). These results were similar to those obtained following VIGS suppression of *PhWD2* when the whole plants were infected (see **Figure 5**). We next determined the expression levels of scent-related genes in the agroinfiltrated petal regions; none were significantly affected by suppression of *PhWD2* (**Figure 7**).

**Figure 8:**
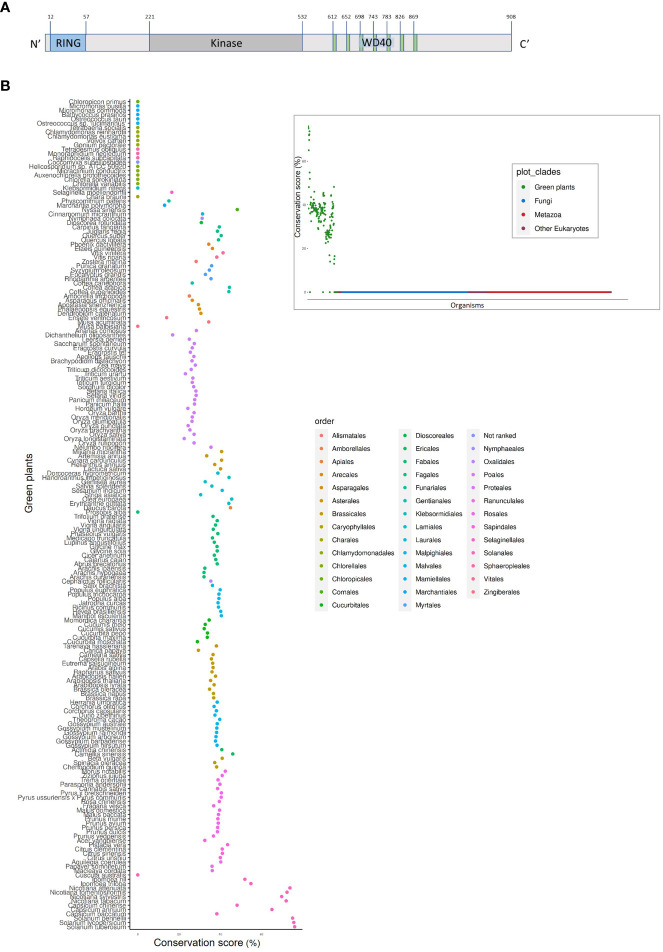
UPPER (PhWD2) protein contains the three-domain structure, which is represented in the majority of the plant taxon. **(A)** Schematic structure of UPPER. Blue, gray and green colors represent the RING, kinase and WD40 domains, respectively. Superscript numbers represent specific amino acids. **(B)** Conservation score (see Materials and methods) of top hits for UPPER across different organisms of the Viridiplantae clade. Inset: Conservation score of top hits for UPPER across all eukaryotes.

PhWD2 has a complex structure (**Figure 8A**); in addition to the seven WD40 repeats at its C-terminal end (as predicted by WDSPdb tool; Ma et al., 2019), it also contains a RING-finger domain at its N terminus and a kinase domain in between, as predicted by the PROSITE tool. No paralogs of PhWD2 were detected by BLASTn/p against the *P. axillaris* genome or *P. hybrida* cv. Mitchell transcriptome (e-value threshold = 0.1). To determine whether PhWD2 has homologs in other organisms, we performed BLASTP against proteomes of 1952 eukaryotic organisms and each best BLASTP bit-score protein (“top hit”) was given a conservation score that represents the percentage of similarity between the two proteins (see Materials and Methods). Interestingly, across all organisms tested, only members of the green plant (Viridiplantae) clade received a conservation score >10, indicating that PhWD2 is unique to this clade (**Figure 8B**, inset); this protein was termed UPPER (Unique Plant PhEnylpropanoid Regulator). Zooming in on the Viridiplantae revealed that UPPER homologs are absent from green algae, as well as from *Prosopis alba*, *Musa balbisiana* and *Cuscuta australis*, whereas representative organisms of the order Solanales had the highest conservation scores (**Figure 8B**). To further assess the conservation level of UPPER among plants, domain analysis was conducted using the NCBI Batch Conserved Domains-Search Tool. Most UPPER homologs in the orders Vitales, Fagales, Myrtales, Cornales, Laurales, Nymphaeales, Gentianales, Asparagales, Poales, Proteales, Asterales, Lamiales, Apiales, Fabales, Oxalidales, Malpighiales, Ericales, Caryophyllales, Rosales and Solanales, and in representative non-vascular plants *Marchantia* and *Selaginella*, contained RING, kinase and WD40 domains (**Supplementary Figure S7**). Top hits in organisms that contained a two-domain structure, e.g. members of Cucurbitales, Brassicales and Sapindales orders, lacked the kinase domain. It would be interesting to explore the evolution of proteins with this three-domain structure and to functionally characterize their roles in plants in general, and in secondary metabolism in particular.

## Discussion

The flux in the phenylpropanoid-biosynthesis pathway switches from the production of pigments to the biosynthesis of floral volatiles in parallel with flower opening. Petal pigmentation occurs at early stages of bud development (Weiss, 2000) and active production of scent starts at anthesis (Spitzer-Rimon et al., 2010; Cna’ani et al., 2017). Another crucial factor in the regulation of many biological processes, especially when studying floral volatiles, is diurnal rhythmicity; many scent-related genes have been shown to be diurnally regulated (Fenske et al., 2015). Methods allowing high-throughput analyses, such as RNA-Seq, have become indispensable tools for research, enabling a broader view of biological processes. To characterize the effect of developmental stage and time of day on transcriptome reorganization with respect to volatile production, we performed RNA-Seq analysis of the petals of flowers at two developmental stages: buds and 1DPA, and at two time points: morning and evening. The bud-to-mature flower transition was accompanied by significant perturbations in the corolla transcriptome: ca. 70% of the transcripts were differently expressed in buds compared to 1DPA petals (**Figure 1**). These results indicate that the physiological processes underlying flower maturation, e.g., opening and scent production, are accompanied by dramatic transcriptomic changes. The secondary metabolism pathway was KEGG-enriched in 1DPA vs. bud DEGs and VOC-biosynthesis genes were upregulated in mature flowers (**Figure 2**), in parallel with the initiation of scent production in petals. Analysis of transcriptomic data for petunia flowers and floral buds at developmental stages comparable to those used in the current study (Patrick et al., 2021) demonstrated similar results, i.e., DEGs with increased transcript levels in the petals of mature flowers vs. buds were enriched in phenylpropanoid-related metabolic processes. We also observed downregulation of the genes involved in anthocyanin biosynthesis in the petals of open flowers compared to buds. Previous studies of petunia and rose petal transcriptomes (Han et al., 2017; Patrick et al., 2021) have also shown that flower maturation is accompanied by a decrease in mRNA levels of a number anthocyanin pathway genes. Transcriptome analyses of buds at several different developmental stages (Yu et al., 2022) revealed the greatest number of DEGs when very young buds (<0.2 cm) were compared to flowers at anthesis (Yu et al., 2022). This also indicates global transcriptome rewriting from growth/cell division and flux of phenylpropanoids toward pigmentation to cell expansion and volatile phenylpropanoid/benzenoid production during flower maturation.

Analysis of the transcriptomic response to time of day in petals revealed that 44% of all transcripts expressed in petunia petals were differentially expressed in the morning vs. evening. These results are in line with studies on leaves of *Arabidopsis thaliana*, *Glycine max*, *Populus trichocarpa* and *Oryza sativa*, where 21–40% of the genes were found to be expressed rhythmically throughout the day (Bläsing et al., 2005; Espinoza et al., 2010; Filichkin et al., 2011; Locke et al., 2018; Nakamichi, 2020). Interestingly, flower developmental stage had an impact on the diurnal pattern of gene expression. For most of the m/e DEGs, temporal changes in expression were observed in only one of the developmental stages tested. About 42% of the petal transcripts revealed m/e changes in expression levels in 1DPA flowers, whereas three-quarters of those genes were not affected by the time of day in buds. In contrast, in floral buds, only ca. 17% of the transcripts were m/e DEGs, and only one-third of those were not affected by time of day in 1DPA petals. This suggests that the response of gene expression to time of day is more prevalent in petunia petals of mature flowers than in floral buds, corresponding to activation of an additional time-dependent physiological process—production of scent—in mature flowers. M/e changes in mRNA levels for the major scent-related genes (**Figure 3B**) support previously documented diurnal oscillations of a number of petunia VOC-biosynthesis genes and regulators, and is in accordance with diurnal oscillations in floral scent emission (Fenske et al., 2015; Cna’ani et al., 2017). *Arabidopsis* research has also demonstrated diurnal rhythmicity in the expression of genes encoding enzymes of the phenylpropanoid pathway, and of the biosynthesis of primary metabolites and amino acids which, in turn, serve as substrates for shikimate and phenylpropanoid pathways (Bläsing et al., 2005; Espinoza et al., 2010; Cervela-Cardona et al., 2021). Diurnal oscillations in gene expression are driven by the circadian clock, and by perceived external factors, mostly light/dark signals (Dodd et al., 2005; Inoue et al., 2017; Oakenfull and Davis, 2017). Organ-specific light input has been shown to affect root–shoot-specific rhythmic properties of the circadian oscillator in *Arabidopsis* (Bordage et al., 2016). Organ-dependent changes in diurnal expression were also shown for the core circadian clock genes in petunia—*PRR*s and *GI*s (Terry et al., 2019).

Using the petunia petal transcriptome generated here, and based on the typical expression patterns of scent-related genes, we were able to identify the WD40 domain protein UPPER, which is involved in the regulation of floral scent. Like most known scent-related genes, *UPPER* was highly expressed in petals of open flowers and in the adaxial epidermis. *UPPER* suppression by VIGS and by transient localized suppression dramatically enhanced VOC emission and accumulation in internal pools, indicating that UPPER is involved in negative regulation of floral scent. Enhancement of VOC production—at the emission and internal pool levels—suggests that UPPER probably affects the biosynthesis of these compounds, and not only the emission machinery.

WD40 proteins are known to participate in numerous cellular processes, including production of specialized metabolites. Their regulation of the phenylpropanoid pathway has been well-studied for *Arabidopsis* TRANSPARENT TESTA GLABRA 1 (TTG1) and its petunia homolog AN11 (de Vetten et al., 1997; Walker et al., 1999; Albert et al., 2014). TTG1 and AN11 positively regulate anthocyanin production, providing a scaffold for protein–protein interactions between bHLH and MYB transcription factors, involved in the regulation of pigment biosynthesis. Similar complexes have been found in many plant systems, e.g., tomato, *Medicago*, corn and strawberry (Carey et al., 2004; Pang et al., 2009; Schaart et al., 2013; Gao et al., 2018). The production of anthocyanins and that of phenylpropanoid volatiles are interlinked processes, as has been demonstrated in carnation, rose and petunia (Zuker et al., 2002; Zvi et al., 2008; Cna’ani et al., 2015). However, the involvement of WD40 proteins in the regulation of phenylpropanoid volatile production in flowers has not been previously documented.

We did not find any significant effect of *UPPER* suppression on transcript levels of the major scent-related genes encoding biosynthesis enzymes, or regulators of the pathway or of the emission machinery. UPPER’s effect might be manifested at the protein level because, in addition to WD40 repeats that are responsible for protein–protein interactions, UPPER contains a RING-finger domain. So far, no WD40 domain has been found to have any intrinsic enzymatic activity; some WD40 proteins contain additional functional or catalytic domains, such as F-BOX and RING domains involved in protein degradation (Stone et al., 2005). Most of the RING domain proteins possess ubiquitination activity and are classified as RING-type E3 ubiquitin ligases—proteins that are responsible for selective targeting of the substrate for ubiquitination, followed by target protein degradation (Stone et al., 2005). In addition to the RING and WD40 domains, UPPER also contains a kinase domain. There is an example of a RING-type E3 ubiquitin ligase that contains RING and kinase domains—the negative regulator of abscisic acid signaling KEEP ON GOING (KEG) (Stone et al., 2006). Nevertheless, UPPER is unique in having all three domains.

Several studies revealed that the kinase activity is important for regulatory role of E3 ubiquitin ligases and it was shown that E3 ubiquitin ligases can work as a part of the multisubunit complex (Lau and Deng, 2012; McNeilly et al., 2018). For example, the RING–WD40 ligase CONSTITUTIVE PHOTOMORPHOGENIC 1 (COP1) can form a complex with the kinase SUPPRESSOR OF PHYA-105 1 (SPA1) which contains WD40 and kinase domains. Together, they interact and phosphorylate photomorphogenesis transcription factors leading them for degradation (Kim et al., 2017; Paik et al., 2019). The presence of the RING, kinase and WD40 domains, commonly involved in E3 ubiquitin ligase functioning, may suggest UPPER’s involvement in protein degradation. Generation of *upper* mutants and analysis of their transcriptome and proteome as well as identification of the proteins interacting with UPPER and studying the roles of the three domains should shed light on its mode of action in floral scent regulation.

## Accession numbers

PhWD1 (Peaxi162Scf00006g00099), PhWD2, UPPER (Peaxi162Scf01003g00015), PhWD3 (Peaxi162Scf00378g00630), DAHP1 (Peaxi162Scf00030g01715), DAHP3 (Peaxi162Scf00381g00086), EPSPS (Peaxi162Scf00959g00022), CS (Peaxi162Scf00747g00122), CM1 (Peaxi162Scf00166g00931), CM2 (Peaxi162Scf00495g00010), ADT1(Peaxi162Scf00114g00001), ADT3 (Peaxi162Scf00002g00514), ADT4 (Peaxi162Scf00147g00613), PAL1 (Peaxi162Scf00858g00215), PAL2 (Peaxi162Scf00123g00096), PAAS, (Peaxi162Scf00561g00021), BSMT1 (Peaxi162Scf00047g01123),BPBT (Peaxi162Scf00007g00011), 4CL (Peaxi162Scf00314g00086), PhBSα (Peaxi162Scf00811g00011), BALDH1 (Peaxi162Scf00017g02715), C4H1 (Peaxi162Scf00556g00035), C4H2 (Peaxi162Scf00390g00225), CFAT (Peaxi162Scf00474g00217), IGS1 (Peaxi162Scf00889g00229), EGS (Peaxi162Scf00020g01714), CNL1 (Peaxi162Scf00294g00411), CHD (Peaxi162Scf00231g00330), KAT1(Peaxi162Scf00052g00819), KAT2 (Peaxi162Scf00047g01234), CCR1 (Peaxi162Scf00332g00433), HCT (Peaxi162Scf00835g00312), ODO1 (Peaxi162Scf00002g00037), EOBI (Peaxi162Scf00129g01231), EOBII (Peaxi162Scf00080g00064), EOBV (Peaxi162Scf00362g00831), PhERF6 (Peaxi162Scf00031g00144), PH4 (Peaxi162Scf00349g00057), PhMYB4 (Peaxi162Scf01221g00042), PhABCG1 (Peaxi162Scf01060g00147), PhABCG11 (Peaxi162Scf01390g00033), PhABCG12 (Peaxi162Scf00004g03212), GID1B1 (Peaxi162Scf01039g00226), GID1B2 (Peaxi162Scf00006g00389), GID1B3GA (Peaxi162Scf00936g00034), GID1C (Peaxi162Scf00434g00079), GA2ox2a (Peaxi162Scf00111g00922), CHI-A (Peaxi162Scf00006g00088), CHIa (Peaxi162Scf00006g00088), F3H (Peaxi162Scf00328g01214), F3'5'H1(Peaxi162Scf00150g00218), F3'5'H2 (Peaxi162Scf00108g00417), F3'H (Peaxi162Scf00201g00243), DFR (Peaxi162Scf00366g00630), ANS (Peaxi162Scf00620g00533), 3GT (Peaxi162Scf00163g00081), 5GT (Peaxi162Scf00378g00113).

## Data availability statement

The data presented in the study are available at NCBI, BioProject accession number PRJNA949605.

## Author contributions

EKS, OS, ELS, YK, DB and SK performed the experiments and analyzed the data. DV and YT analyzed the data. EKS, OS and AV wrote the manuscript. AV supervised the study. All authors contributed to the article and approved the submitted version.
