# Supplementary material for: Developmental and temporal changes in petunia petal transcriptome reveal scent-repressing plant-specific RING–kinase–WD40 protein
Source: Front Plant Sci. 2023 Jun 8;14:1180899. doi: 10.3389/fpls.2023.1180899 (PMC10286513; doi:10.3389/fpls.2023.1180899)
Supplement: Supplementary file 6 [file DataSheet_1.pdf]

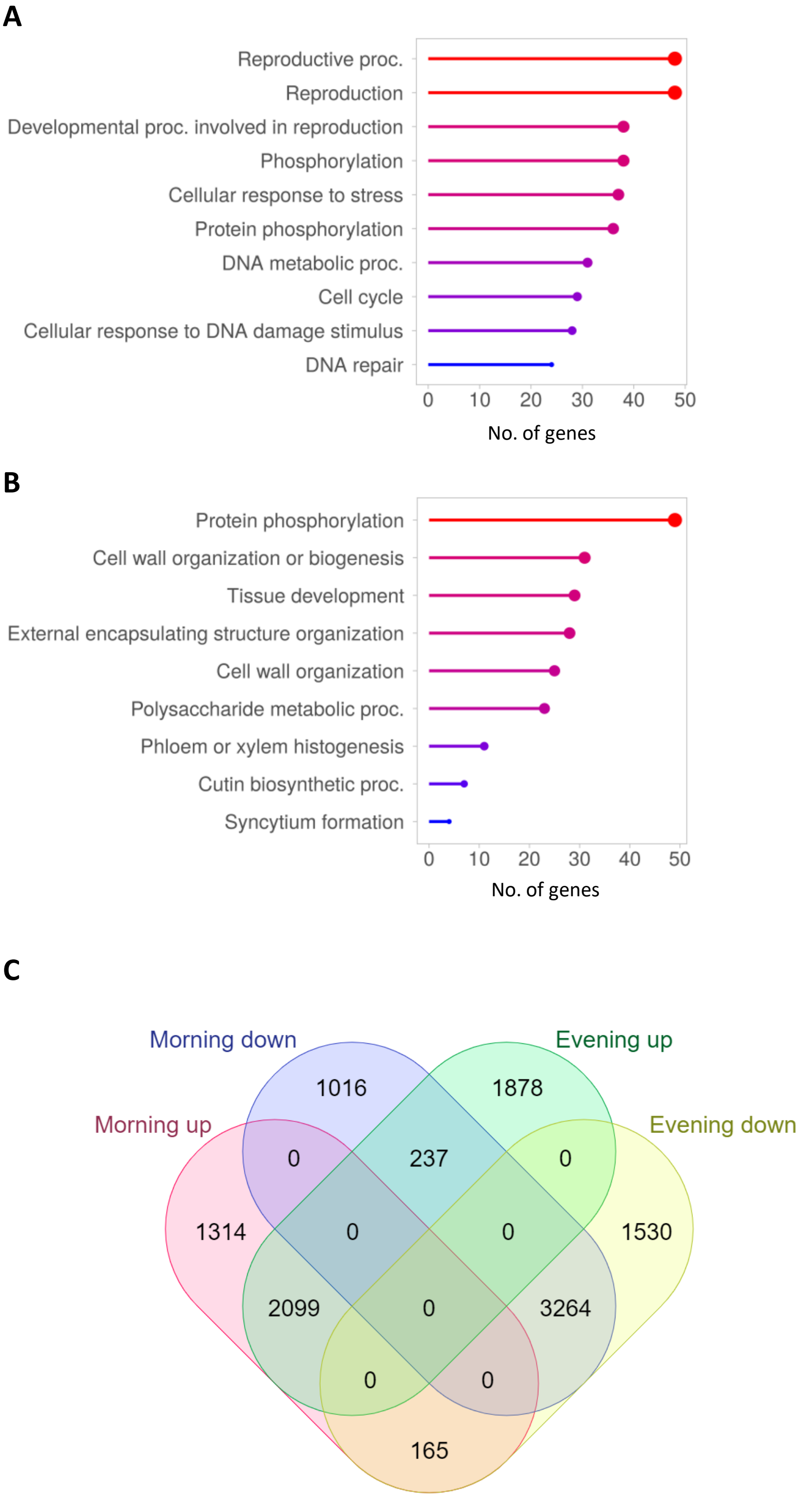

Supplementary Figure 1. Differentially expressed genes (DEGs) for 1 day postanthesis (1DPA) flowers vs. buds comparison. **(A, B)** GO Biological Processes enrichment for 1DPA vs. bud DEGs exclusively expressed in **(A)** 1DPA petals and **(B)** buds. **(C)** Venn diagram showing numbers of upregulated and downregulated 1DPA vs. bud DEGs in the morning and in the evening.

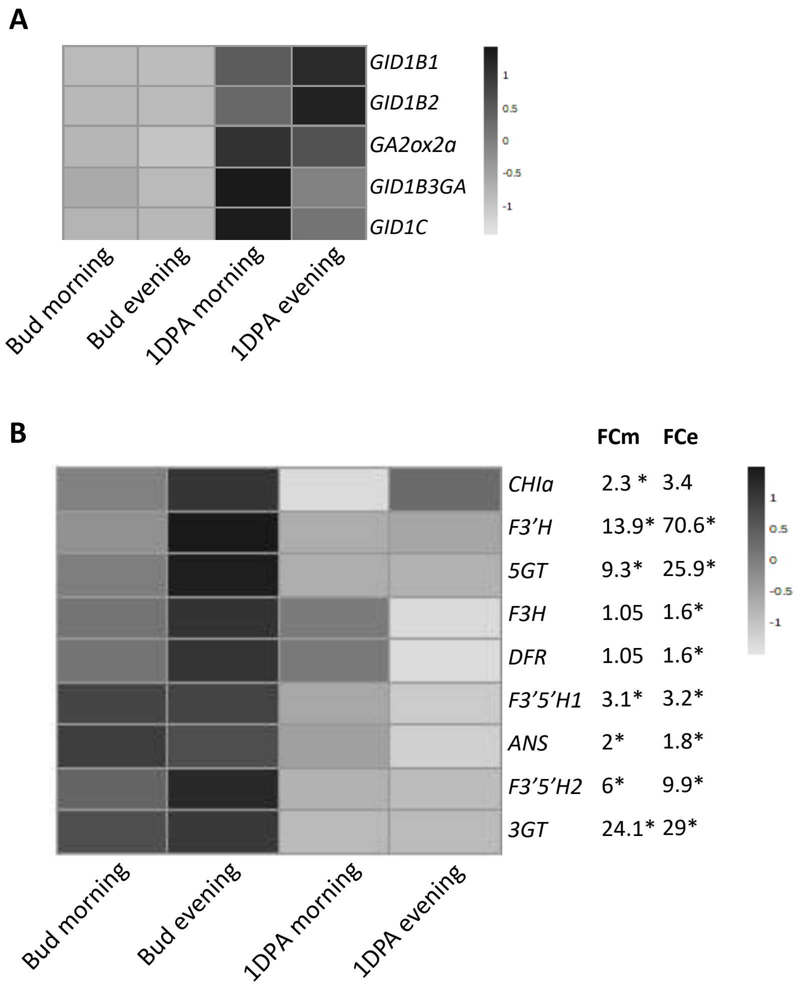

Supplementary Figure 2. Heatmaps representing the expression levels of GA-related genes (A) and anthocyanin biosynthesis genes (B) in petunia petals. Transcript levels in 1 day postanthesis (1DPA) flowers and buds in the morning (1000 h) and in the evening (1900 h) are presented. Average normalized counts for three biological replicates are plotted; scaling was applied for each separate gene. FC – fold change in average normalized counts for bud vs. 1DPA comparison in the morning (FCm) and in the evening (FCe). *Differentially expressed genes ($P \leq 0.05$, log2FC $\geq$ 0.585, normalized count $\geq$ 30). Abbreviations: CHI, chalcone isomerase; F3H, flavanone 3-hydroxylase; F3′5′H, flavonoid 3′,5′-hydroxylase; F3′H, flavonoid 3′-hydroxylase; DFR, dihydroflavonol 4-reductase; ANS, anthocyanin synthase; 3GT, 3-glucosyl transferase; 5GT, 5-glucosyl transferase.

A

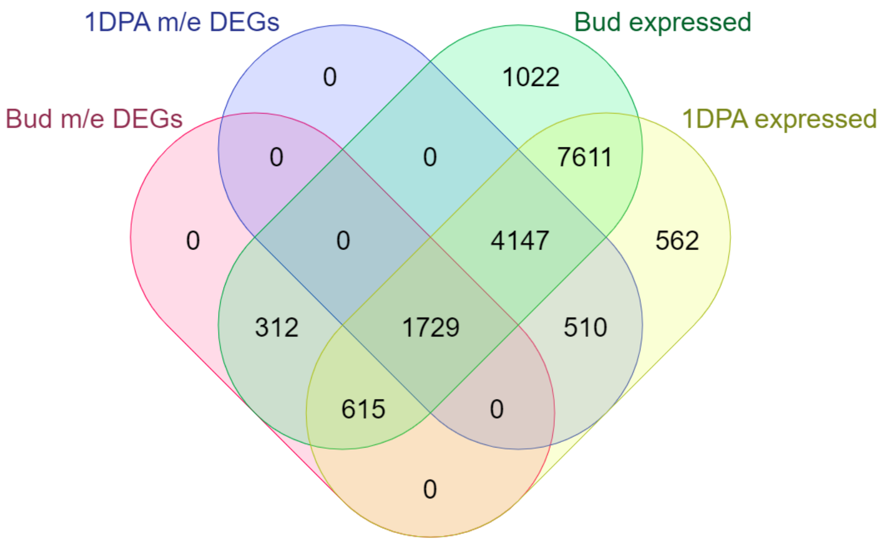

B

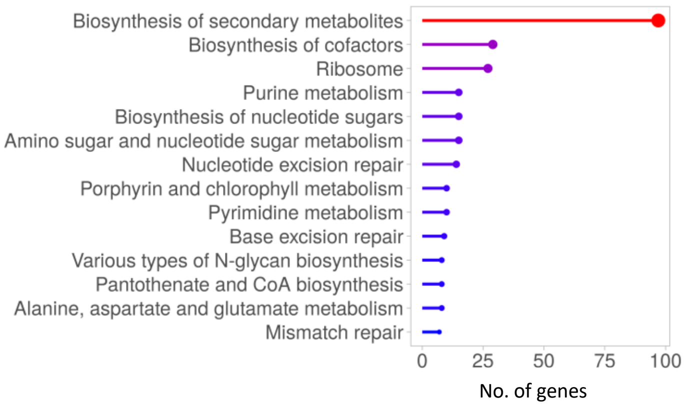

Supplementary Figure 3. Differentially expressed genes (DEGs) for morning vs. evening (m/e) comparison. **(A)** Venn diagram showing numbers of m/e DEGs expressed in buds and/or 1 day postanthesis (1DPA) petals. **(B)** KEGG enrichment for 1DPA m/e DEGs that are more highly expressed in buds than in 1DPA petals.

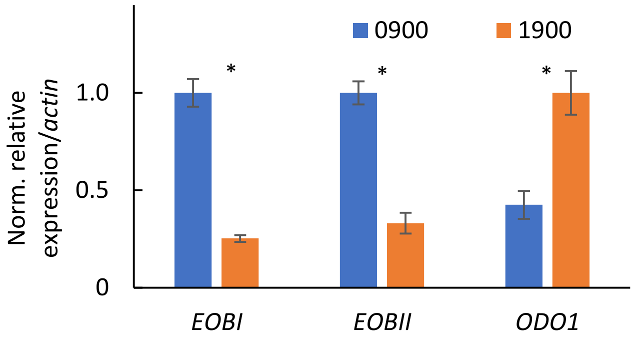

Supplementary Figure 4. Expression levels of selected transcriptional regulators of floral scent. Petals of Petunia cv. Mitchell 1 day postanthesis (1DPA) flowers were collected in the morning (0900 h) and in the evening (1900 h) for RNA extraction followed by qRT-PCR. *Actin* was used as an internal reference gene. Relative expression was normalized to the maximum for each target gene. Data are means ± SEM (n = 4). Significance of differences was calculated by Student's t-test: $^*P \leq 0.05$.

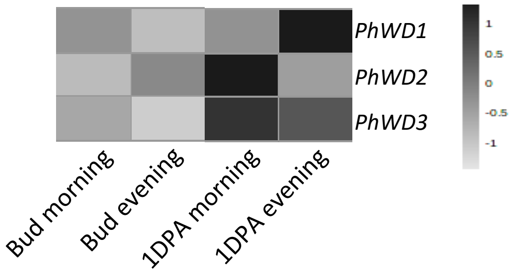

Supplementary Figure 5. Heatmap representing the expression levels of *PhWDs* in petunia petals, based on RNA-Seq. Transcript levels in 1 day postanthesis (1DPA) flowers and buds in the morning (1000 h) and in the evening (1900 h) are presented. Average normalized counts for three biological replicates are plotted; scaling was applied for each separate gene.

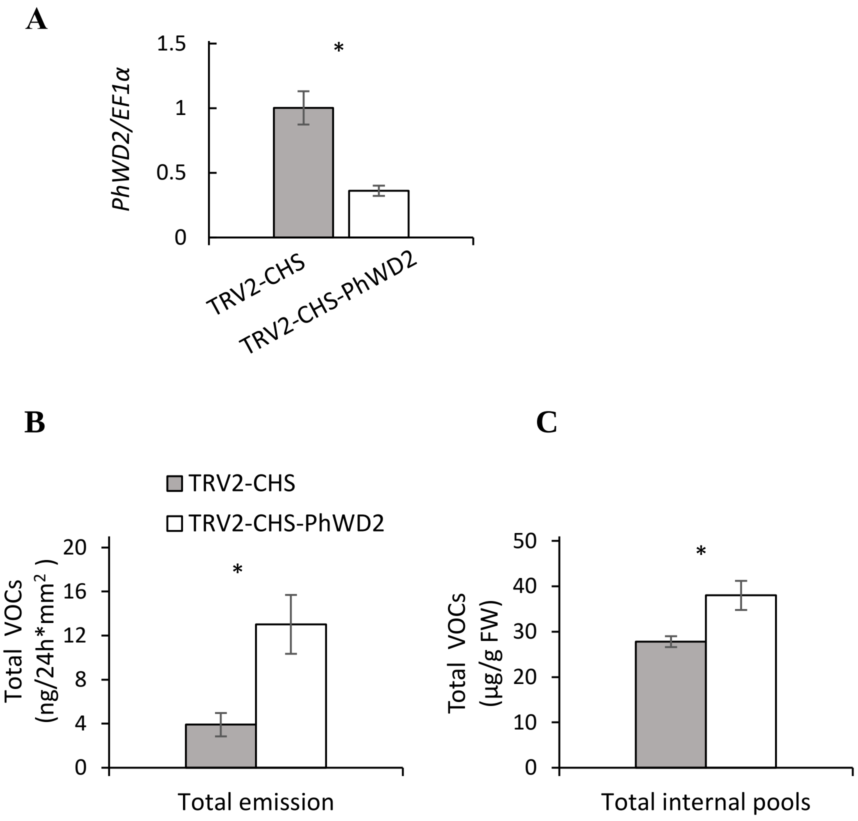

Supplementary Figure 6. Transient localized suppression of *PhWD2* in petunia line 720 flowers leads to increased volatile organic compound (VOC) production. Petals of flowers at anthesis were infiltrated with a suspension of *Agrobacterium* carrying TRV2-CHS-PhWD2 or TRV2-CHS (control). Inoculated petal areas were used for analysis. **(A)** Normalized relative expression of *PhWD2*, measured by qRT-PCR. Samples were collected at 2000 h. *EF1α* was used as an internal reference gene. Expression levels of *PhDW2* were normalized to those in control. n = 4. **(B)** Total emitted volatiles, collected by localized headspace followed by GC-MS analysis (n = 6–8). **(C)** Total level of VOCs accumulated in internal pools (n = 4). Data are means ± SEM. Significance of differences between treatments was calculated using Student's t-test: $*P \leq 0.05$.

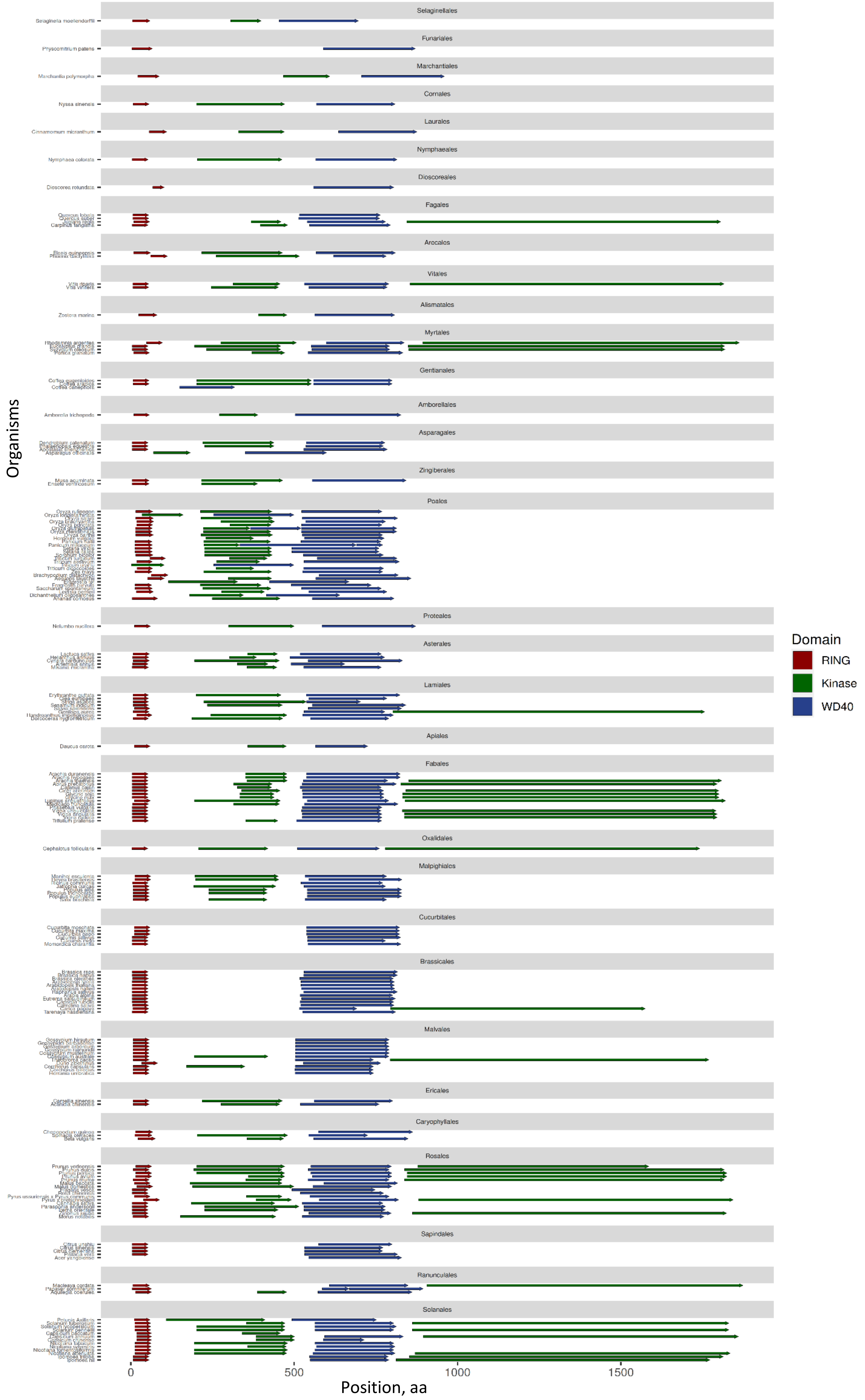

Supplementary Figure 7. Domain analysis of UPPER homologs across green plants.

Supplementary Table S1. Primers used for amplification of the fragments for cloning into pTRV2 (5’ -> 3’). Sizes of the obtained amplicons are designated

Gene	Forward primer	Reverse primer	Amplicon
PhWD1	AATCTAGATTGACATTCGAAGCACAACA	AAGAGCTCGCATGTCTTTCAGTCCCGATA	254 bp
PhWD2	ATGGATCCAAGTGATTGAGGCAGCAACA	ATGAATTCTCCCTCATACCAAGTTCAAGGAA	252 bp
PhWD3	ATGAATTCCAGGCAACGGAGATATCAAC	ATGGATCCATGAACATGGTGCTTTTCTTATGA	227 bp

Supplementary Table S2. Primers used for qRT-PCR

	**qRT-PCR primers (5'->3')**	
**Gene**	**Forward primer**	**Reverse primer**
EF1α	TGAGATTCTGCGTGGATGAA	CCCATCAAGCAACTTGGACT
Actin	TGCTGATCGTATGAGCAAGGAA	GGTGGAGCAACAACCTTAATCTTC
PhWD1	TGTTGCTCCTTTGTGTGGACATG	CCGGACATCCCATATTCTGCA
PhWD2	ATAGATTGAGCGACGGCGAG	TCCCAGCTATGTCGCTAACC
PhWD3	CCACTTCCGGTTCCAATGGCC	GGCAATCGTCTTTCCGTTGGAGG
DAHPS	TTGAGGGCTTTTGCTACTGG	GTCCAGAACTCGGTGGTTGT
EPSPS	GCGGAAAACCTTCCCTAACT	GCCCTTCTGAACTGAAATGG
PAL2	TGCTAATGGTGAACTTCATCCA	TGACATTCTTCTCACTTTCACCA
BPBT	TGTTGAAGGGTGATGCTCAA	GGATTTGGCATTTCAAACAAA
C4H	CCAGGAGTGC AAGTGACTGA	CTTCCAGTGA GCAGGGTTGT
IGS	CCACGTCAAAAGAGTGAGCA	CCAGTGGTTTTCTCCCAAGA
EOBI	TTCTCCATCTTCATACACTGGAAATA	GCTAGGCAGCTAGATTACTGATT
EOBII	CCATTGATCAGACCTACTCTCCCAC	GGACCAGATGTCTTCCATGCTCC
ODOI	ACCAACC`TACCAACCAACCA	ATGATGACCCCTCCAACAAG
ABCG1	CCCGTGATCTTGGCTACTACTGG	GAACCTCTTGCCTGAACTGAAGC
PH4	TGATCCTTTCATGGCTTCATC	GCTACAACATCATCAAGAGAAAGAAT

Supplementary Dataset S1

Genes expressed in petals of petunia floral buds and 1 day postanthesis (1DPA) flowers, GO terms.

Supplementary Dataset S2

Differentially expressed genes (DEGs) in 1 day postanthesis (1DPA) flowers vs. bud comparison.

Supplementary Dataset S3

Groups of differentially expressed genes (DEGs) in 1 day postanthesis (1DPA) flowers vs. bud, KEGG enrichment

Supplementary Dataset S4

Differentially expressed genes (DEGs) for morning vs. evening comparison, KEGG enrichment.

Supplementary Dataset S5

Transcripts encoding WD40 proteins that are upregulated in 1 day postanthesis (1DPA) flowers vs. buds.
